# Simultaneous Determination of Glucose and Cholesterol in Milk Samples by Means of a Screen-Printed Biosensor and Artificial Neural Networks

**DOI:** 10.3390/bioengineering13030274

**Published:** 2026-02-27

**Authors:** Jessica Torres-Gámez, José A. Rodríguez, María Elena Páez-Hernández, Carlos A. Galán-Vidal

**Affiliations:** Área Académica de Química, Universidad Autónoma del Estado de Hidalgo, Carretera Pachuca-Tulancingo Km. 4.5, Mineral de la Reforma 42084, Hidalgo, Mexico; jessicatorresgamez17@gmail.com (J.T.-G.); josear@uaeh.edu.mx (J.A.R.); paezh@uaeh.edu.mx (M.E.P.-H.)

**Keywords:** flavored milk, glucose, cholesterol, artificial neural networks, voltammetric biosensor

## Abstract

In the present work, a bienzymatic voltammetric biosensor is reported for the simultaneous quantification of glucose and cholesterol in flavored milk samples with a single device. The biosensor is based on a carbon screen printing electrode on which paper disks impregnated with glucose oxidase and cholesterol oxidase, as well as ferricyanide as a mediator, are deposited. Linear voltammetry combined with an artificial neural network methodology were successfully used for the determinations, showing excellent agreement with the spectrophotometric reference method.

## 1. Introduction

Dramatic lifestyle changes in recent years have caused physical inactivity, as well as a rise in sugar and fat intake, and have compromised the health of a significant percentage of the population [1,2]. In this sense, the determination of glucose and cholesterol is particularly important because abnormal levels of these biomolecules in biological fluids are associated with several diseases, including diabetes and hypertension, whose prevalence is increasing and which are currently considered among the 10 leading causes of death [2,3,4]. In addition to quantifying glucose and cholesterol in biological fluids, it is also important to quantify these substances in food, to assess the risk associated with their consumption, as well as in the production and quality control of food [5].

The relevance of glucose and cholesterol determination has promoted the design of a wide range of methods for their quantification in foods and biological fluids such as spectrophotometry, liquid chromatography, gas chromatography, and capillary electrophoresis [6,7]. Although many of the reported methodologies are well established and satisfy the requirements for samples of food and clinical interest, there are still some disadvantages to their implementation that make them expensive, including exhaustive sample pretreatment, long analysis times, low sensitivity and selectivity, as well as the need for expensive instrumentation [8]. Electrochemical methods, particularly electrochemical biosensors, constitute an attractive alternative, as they require inexpensive instrumentation, are easy to use, and decentralized analysis can be implemented while maintaining competitive analytical performance compared to expensive instrumental analysis methods [9].

Efficient analytical alternatives for the individual quantification of glucose or cholesterol in biological and food samples have been reported; nevertheless, reports on their simultaneous determination are scarce. Among them, these methods have been reported: Fourier transform infrared spectroscopy (FTIR) in blood serum using partial least squares regression (PLS) [10], attenuated total reflectance FTIR in whole blood using PLS [11], Fourier transform Raman spectroscopy (FT-Raman) in whole blood using PLS [12], UV-Vis spectrophotometry in blood serum using PLS and artificial neural networks (ANNs) [13], reflective optical fiber surface plasmon resonance (SPR) sensor in standard samples [14], colorimetry in whole blood using linear calibration curves obtained from color intensity images of a microfluidic cell with a paper pad [15] optical fiber SPR sensor in standard samples [16], colorimetry in whole blood using linear calibration curves and by comparison with color cards from color intensity obtained in an analytical device based on paper pads [17] and colorimetry of fluorescence obtained on a paper pad-based analytical device in whole blood using linear calibration curves [18]. Most of these reports use relatively unaffordable instrumentation that could scarcely be implemented for decentralized analysis.

Reports regarding electrochemical methodologies for the simultaneous determination of cholesterol and glucose include an amperometric biosensor based on a polythionine-modified glassy carbon electrode on which Au nanoparticles are deposited to immobilize glucose oxidase or cholesterol oxidase enzymes, for the analysis of mouse serum [19], an amperometric biosensor array based on a Ti/Au film on which polyaniline (PANI) is electropolymerized and subsequently Pt nanoparticles are deposited to finally immobilize glucose oxidase or cholesterol oxidase for the analysis of doped serum samples [20], a voltammetric sensor based on a Ti_3_C_2_/chitosan/Cu_2_O film for the analysis of diluted blood serum samples [9] and amperometric biosensors based on a glassy carbon electrode modified with polydopamine-coated magnetite nanoparticles and immobilizing glucose oxidase or cholesterol oxidase [21]. In these works, the devices are coupled to multichannel electrochemical analyzers, where the independent signals from each biosensor are obtained and processed using individual calibration curves for each analyte. The above examples also make evident the need to quantify cholesterol and glucose in food, such as milk, as well as the application of multivariate analysis to make processes more efficient.

This paper reports the development of a voltammetric biosensor for the simultaneous determination of glucose and cholesterol using a single device constructed from a paper circle containing cholesterol oxidase, glucose oxidase, and a mediator placed on carbon screen-printed electrodes. The biosensor incorporates two enzymes to simultaneously quantify glucose and cholesterol with a single device instead of the conventional setup of using a separate biosensor for each analyte to quantify them independently or in parallel via an electronic tongue.

## 2. Materials and Methods

### 2.1. Materials and Instrumentation

All reagents used were of analytical grade. Glucose oxidase (GOx, EC 1.1.3.4, type X-S from *Aspergillus niger*), cholesterol oxidase (ChOx, EC 1.1.3.6 from *Streptomyces* sp.), glucose, potassium ferricyanide (III), Triton X-100 and potassium phosphate were purchased from Sigma-Aldrich (St. Louis, MO, USA). Cholesterol was purchased from Research Organics (Cleveland, OH, USA). Potassium phosphate monobasic, potassium chloride, methanol and ethanol were purchased form J.T Baker (Phillipsburg, NJ, USA). The glucose enzymatic reagent was composed of glucose oxidase (GOx, 1500 U L^−1^), peroxidase (POD, 1000 U/L), phenol and 4-aminoantipyrine (4-AP). The cholesterol enzymatic reagent was composed of cholesterol esterase (ChE, 1000 U L^−1^), cholesterol oxidase (ChOx, 3000 U L^−1^), phenol and 4-AP. Enzymatic reagents were supplied by Spinreact (Girona, Spain).

A cholesterol stock solution (0.05 mol L^−1^) was prepared in ethanol using vortex mixing to obtain a homogeneous solution. Then Triton X-100 (1%, *v*/*v*) was added to enhance cholesterol solubility in aqueous solutions [22]. A 0.1 mol L^−1^ phosphate buffer solution (PBS) (pH 7) was prepared using K_2_HPO_4_, KH_2_PO_4_, and KCl. All solutions were prepared using deionized water from a Milli-Q water purification system (Millipore, Bedford, MA, USA).

All electrochemical characterizations and measurements were performed using a PGSTAT 30 AUTOLAB electrochemical system (Ecochemie, Methrohm Autolab, Utrecht, The Netherlands). Screen-printed carbon electrodes (SPCE, DRP-110) and the boxed connector for screen-printed electrodes (DRP-DSC) were purchased from DropSens (Asturias, Spain). The planar electrodes are designed using a working electrode (4 mm diameter) and a counter electrode, both made of carbon, while Ag/AgCl serve as the pseudo-reference electrode.

### 2.2. Construction of the Bienzymatic Biosensor

**Figure 1:**
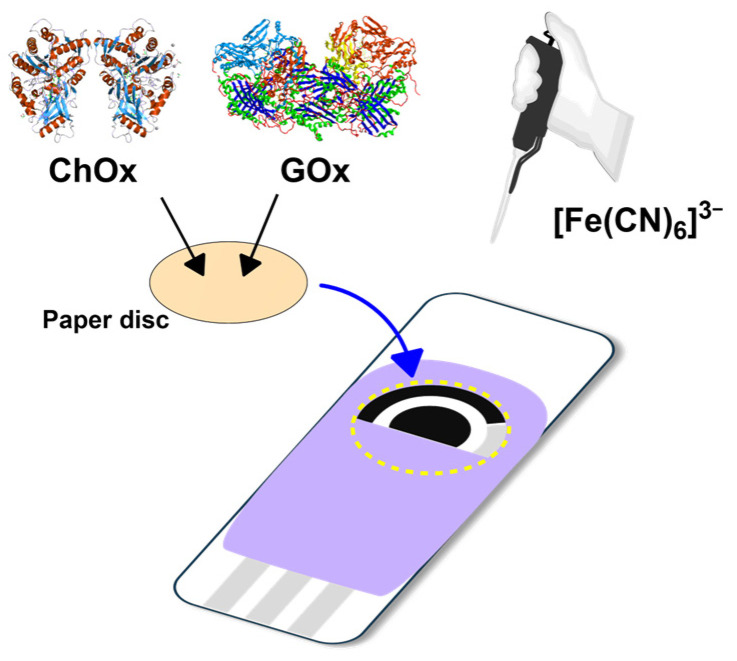
Schematic illustration of the construction process of the bienzymatic biosensor.

Grade 41 filter paper (Whatman Asia Pacific Pte Ltd., Singapore) was cut into round disks with a diameter of 10 mm using a paper hole puncher [23]. Then, 20 μL of enzymatic solution (60 U mL^−1^ of GOx and 20 U mL^−1^ of ChOx in 0.1 mol L^−1^ PBS, pH 7) were carefully added to each paper disk and dried at room temperature (25 °C). These paper disks, loaded with both enzymes, were used for glucose–cholesterol analyses and stored at 4 °C for stability. The paper disk with the immobilized enzymes was carefully placed on top of the SPCE strip to completely cover the working, counter, and reference electrodes before each measurement. A total of 10 μL of potassium ferricyanide (0.01 mol L^−1^) solution was added onto the paper disk to ensure adequate contact with the SPCE (Figure 1). Afterward, electrochemical experiments were performed with the addition of 20 μL of a solution containing glucose and cholesterol.

### 2.3. Multivariate Determination

**Figure 2:**
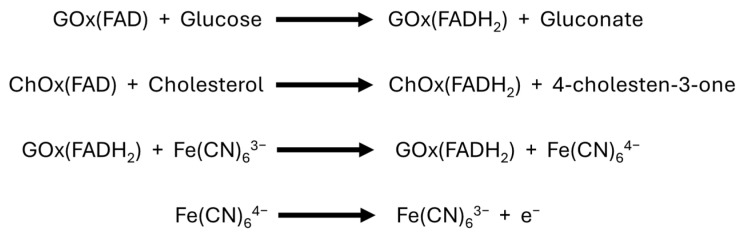
Reactions involved in the simultaneous detection mechanism of glucose and cholesterol with the bienzymatic biosensor.

According to the design of the bienzymatic device, glucose is determined by its reaction with glucose oxidase, whose coenzyme FAD is reduced to FADH_2_ and then reoxidized by ferricyanide (mediator), producing ferrocyanide. This ferrocyanide is finally oxidized on the electrode surface, generating a current proportional to the glucose concentration as a function of the applied potential. Similarly, cholesterol reacts with cholesterol oxidase, generating a current proportional to the cholesterol concentration due to the presence of the mediator (Figure 2). Although the current generated by both analytes is due to the oxidation of the same reduction product (ferrocyanide), the analytical sensitivity differs, making it possible to differentiate the contribution of each analyte by multivariate techniques [13].

For the simultaneous study, standard solutions were prepared by mixing different concentrations of glucose and cholesterol in PBS at pH 7 (Table 1). Samples 1–18 were designed to obtain the calibration and validation set. The concentrations were established through an experimental central composite design (CCD) for two factors, with two central points in the concentration range of 0.59 to 3.41 mmol L^−1^ for glucose and 0.15 to 0.44 mmol L^−1^ for cholesterol. Voltammetric experiments were carried out from open circuit potential to +0.70 V; each discrete signal was composed of 242 data. Data analysis was performed using MATLAB version 9.2 (The Math-Works, Natick, MA, USA) using the NN-toolbox.

### 2.4. Sample Analysis

Six samples of flavored milk were purchased at local markets in Pachuca, Hidalgo, Mexico. For analysis, the milk samples needed pretreatment to precipitate the proteins. To begin this process, 2 mL of the milk sample was measured into 15 mL polypropylene centrifuge tubes. Next, 6 mL of methanol was added to each tube, and the mixture was vortexed for 1 min and centrifuged at 3500× *g* rpm for 10 min. A 500 μL aliquot of the supernatant was transferred into a 10 mL volumetric flask and diluted to volume with a phosphate buffer solution at pH 7 [24].

### 2.5. Analysis of Flavored Milk Samples with the Reference Method

Quantification of glucose and cholesterol in the flavored milk samples was carried out individually using colorimetric commercial assay kits supplied by Spinreact. Glucose or cholesterol reacts with glucose oxidase or cholesterol oxidase, respectively, to produce H_2_O_2_ which reacts with 4-AP, phenol and peroxidase to generate a colored compound whose absorbance is proportional to the concentration of each analyte [13].

Sample preparation and analytical procedures were performed in accordance with the manufacturer’s guidelines. Working solutions were prepared by combining 20 μL of the sample with 2 mL of the respective reagent for either glucose or cholesterol determination. A blank solution was prepared using 2 mL of reagent alone, whereas the standard solution consisted of 20 μL of glucose or cholesterol stock solution mixed with 1 mL of reagent.

All absorbance measurements were conducted using a Perkin Elmer Lambda 40 UV-Vis spectrophotometer (Perkin Elmer, Madrid, Spain) at a wavelength of 550 nm.

## 3. Results

In the first step, the electrochemical behavior of glucose and cholesterol was studied using a linear sweep voltammetry experiment with a SPCE. These experiments were carried out at a scan rate of 0.1 V s^−1^. The electrochemical signal found at 0.20 V corresponds to the oxidation of ferrocyanide produced by the process of regenerating the active site of the enzymes [19,25].

**Figure 3:**
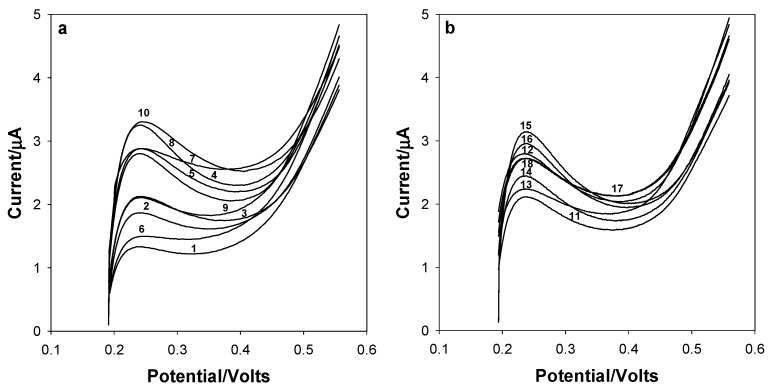
Linear sweep voltammograms of solutions used in (**a**) the calibration set (samples 1–10 of Table 1) and (**b**) the internal validation set (samples 11–18 of Table 1).

Because cholesterol- and glucose-associated signals are found at the same potential, it is necessary to use multivariate methods for the determination of both analytes. As a first approach, the PLS method was applied; however, this model revealed behavior outside of linearity. Based on these results, it was decided to use an ANN model to generate a nonlinear regression model. Therefore, linear sweep voltammograms of glucose and cholesterol solutions, as described in Table 1, were recorded at the SPCE. Figure 3a shows the linear sweep voltammograms obtained for samples 1–10 of Table 1, which were used to obtain the calibration model. Additionally, Figure 3b displays the linear sweep voltammograms obtained for samples 11–18 in Table 1, which were utilized in the internal validation model. All voltammograms were centered.

The artificial neural network (ANN) model was developed to estimate the concentrations of glucose and cholesterol. To determine the most effective neural network configuration, several architectures were evaluated for constructing and validating the predictive model. This included an input layer, a hidden layer, and an output layer. The number of neurons in the input layer corresponded to the number of independent variables introduced into the model—342 parameters derived from the voltammetric signals—while the output layer comprised two neurons, each representing one of the target analytes: glucose and cholesterol.

The optimal quantity of neurons in the hidden layer was established by evaluating multiple ANN architectures through the following procedure: initially, a network with N hidden neurons was constructed. The training algorithm and transfer function were then specified, and the network was trained using the calibration dataset, which was partitioned into 70% for training, 15% for validation, and 15% for testing. The evaluation of the model’s performance was conducted utilizing a distinct validation dataset (independent from the calibration set), and root mean square error (RMSE) values were calculated for both the calibration and validation phases.

This process was repeated for different combinations of training algorithms and transfer functions. The optimal configuration defined by the number of neurons in the hidden layer, the transfer function, and the training method were selected based on the lowest values of the mean squared error (MSE), relative error percentage (RE%), prediction relative error percentage (REP%), and RMSE for both analytes [26,27].

The methodology was applied to determine the most suitable network architecture for the resolution of measured signals. Therefore, the best network model was obtained using 342-93-2 architecture, that is, 342 neurons in the input layer, 93 in the hidden layer and two in the output layer. The most appropriate algorithm in the training stage was that of Levenberg–Marquardt [26,27,28]. The optimized parameters and the estimated errors from the ANN methodology are presented in Table 2 and Table 3, respectively.

**Figure 4:**
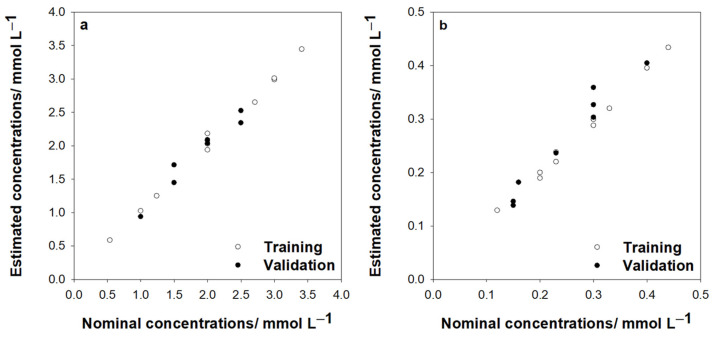
Comparison between the estimated values obtained by ANNs and the nominal concentrations using the calibration set and validation set for (**a**) glucose and (**b**) cholesterol.

Figure 4a,b display the estimated concentrations compared to the nominal concentrations for glucose and cholesterol, respectively. Table 4 shows the calculated regression lines between the obtained and the expected concentration values for the two analytes. In all cases, comparison lines agree with ideal comparison lines of 1 slope and zero intercept [29]. With this comparison and %REP values in the range between 2.88% and 7.19%, it is possible to accept the method as accurate. Thus, the ANN method is suitable as a calibration model for quantifying glucose and cholesterol.

**Figure 5:**
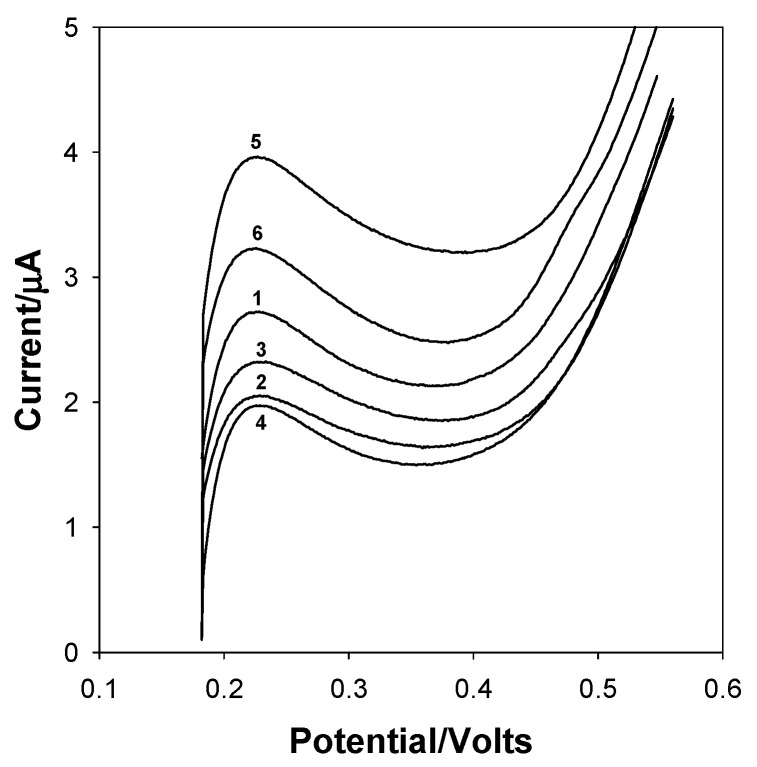
Linear sweep voltammograms of flavored milk samples.

Finally, concentrations of glucose and cholesterol in the flavored milk were calculated. The linear sweep voltammograms used are shown in Figure 5; the data were centered. The results obtained are summarized in Table 5, which shows the estimated concentration by the ANN modeling and its comparison with the reference values. Samples 1 and 4 correspond to chocolate milk, 2 and 5 to strawberry milk, and 3 and 6 to vanilla milk. All samples correspond to different brands.

As can be seen, glucose and cholesterol determinations reached good accuracy. To determine whether there are significant differences between the two methods, a paired t-test was performed. In this test, a t-value of 2.41 was obtained for glucose, and 0.31 for cholesterol, with a t-critical value of 2.57 (α = 0.05). Since the obtained t-value is less than the t-critical for both analytes, there are no significant differences between the two methods. Therefore, the linear sweep voltammetry with ANNs could be an alternative method for the simultaneous determination of glucose and cholesterol.

## 4. Conclusions

The combination of Linear Sweep Voltammetry (LSV) and Artificial Neural Networks (ANNs) proved to be an effective approach for the quantification of glucose and cholesterol in flavored milk samples, employing a straightforward extraction protocol. Furthermore, the results obtained using this electrochemical chemometric strategy showed excellent concordance with those derived from the reference spectrophotometric method, demonstrating the reliability and accuracy of the proposed methodology. The developed biosensor could be applied to different types of milk and used to classify milk quality; to generalize its use to other types of samples, it is necessary to refine the training with a larger number of matrices.

## Figures and Tables

**Table 1:** Concentration values of glucose and cholesterol in pH 7 buffer solution for calibration and validation set.

Experiment Number	Glucose (mmol L^−1^)	Cholesterol (mmol L^−1^)
1	0.54	0.30
2	1.00	0.20
3	1.24	0.23
4	2.00	0.12
5	2.00	0.33
6	2.00	0.44
7	2.71	0.23
8	3.00	0.20
9	3.00	0.40
10	3.41	0.30
11	1.00	0.40
12	1.50	0.30
13	1.50	0.15
14	2.00	0.16
15	2.00	0.30
16	2.00	0.23
17	2.50	0.15
18	2.50	0.30

**Table 2:** Optimized parameters of ANNs.

Parameter	Value
Architecture	342-93-2
Number of iterations	180
Hidden layer transfer function	Tansing
RMSETr (mmol L^−1^) ^1^	0.4072
RMSEM (mmol L^−1^) ^2^	0.5364
RMSET (mmol L^−1^) ^3^	0.3006

^1^ Training for calibration set; ^2^ Monitoring for calibration set; ^3^ Test for calibration set.

**Table 3:** Errors estimated for simultaneous determination of glucose and cholesterol by ANNs.

	Glucose	Cholesterol
RMSEC (mmol L^−1^) ^1^	0.67	0.08
REP (%) ^1^	2.88	3.09
RMSEP (mmol L^−1^) ^2^	1.07	0.24
REP (%) ^2^	5.17	7.19

^1^ For calibration set; ^2^ For prediction set.

**Table 4:** Estimated correlation between the nominal values in training and validation sets employing the final ANN configuration.

		Slope	Intercept (mmol L^−1^)	r^2^
Training	Glucose	0.98 ± 0.02	0.05 ± 0.06	0.9949
	Cholesterol	0.97 ± 0.02	0.01 ± 0.01	0.9947
Validation	Glucose	0.97 ± 0.09	0.08 ± 0.17	0.9528
	Cholesterol	1.08 ± 0.09	−0.01 ± 0.02	0.9568

**Table 5:** Results obtained in the determination of glucose and cholesterol in flavored milk samples.

	Glucose (g/100 g)			Cholesterol (mg/100 g)		
Sample	Obtained	Reference ^a^	REP (%)	Obtained	Reference ^a^	REP (%)
1	7.5 ± 0.5	7.7 ± 0.1	2.60	13.8 ± 1.1	13.7 ± 0.2	0.73
2	6.5 ± 0.9	6.5 ± 0.7	0.00	10.3 ± 1.1	9.9 ± 0.8	4.04
3	7.2 ± 1.3	7.3 ± 0.1	1.37	3.6 ± 0.2	4.0 ± 0.4	10.00
4	7.5 ± 0.5	8.2 ± 0.2	8.54	15.8 ± 0.4	15.1 ± 0.1	4.63
5	10.5 ± 0.1	10.6 ± 0.2	0.94	9.0 ± 0.4	8.5 ± 0.6	5.88
6	8.0 ± 0.3	8.2 ± 0.8	2.44	7.7 ± 0.5	8.6 ± 0.2	10.47

^a^ Spectrophotometric commercial assay kits.

## Data Availability

The raw data supporting the conclusions of this article will be made available by the authors on request.

## Abbreviations

The following abbreviations are used in this manuscript:

FTIR	Fourier transform infrared spectroscopy
PLS	Partial least squares regression
FT-Raman	Fourier transform Raman spectroscopy
ANN	Artificial neural network
SPR	Surface plasmon resonance
PANI	Polyaniline
GOx	Glucose oxidase
ChOx	Cholesterol oxidase
POD	Peroxidase
4-AP	4-aminoantipyrine
ChE	Cholesterol esterase
SPCE	Screen-printed carbon electrode
FAD	Flavin Adenine Dinucleotide (oxided form)
FADH2	Flavin Adenine Dinucleotide (reduced form)
CCD	Central composite design
RMSE	Root mean square error
MSE	Mean squared error
RE%	Relative error percentage (),
REP%	Prediction relative error percentage
